# Tubule-Specific RGC-32 Knockout Exhibits Direct and Progressive Aggravating Activity Against Renal Function in an Ischemia–Reperfusion Mouse Model

**DOI:** 10.3390/biology15171535

**Published:** 2026-09-04

**Authors:** Yan Gong, Dan Feng, Jing Zhang, Mengying Li, Wenyan Huang

**Affiliations:** Department of Nephrology, Shanghai Children’s Hospital, School of Medicine, Shanghai Jiao Tong University, Shanghai 200062, China

**Keywords:** acute kidney injury, chronic kidney disease, RGC-32, renal tubule-specific knockout mouse

## Abstract

Although the prevalence of acute kidney injury and chronic kidney disease remains high and effective therapeutic targets remain scarce, significant progress has been made in recent years across the following major directions: G2/M phase cell cycle arrest, DNA damage, mitochondrial dysfunction, hypoxia-inducible factor signaling, dysregulated autophagy, and epigenetic alterations. RGC-32 is abundantly expressed in all tubular segments of normal renal tissues and is primarily localized to the cytoplasm and perinuclear region of renal tubular epithelial cells. Moreover, RGC-32 is involved in cell cycle regulation as well as cell proliferation and differentiation. To explore the functional role of RGC-32 in renal repair after acute ischemia–reperfusion injury, we utilized CRISPR-Cas9 technology combined with Cre/loxP recombination to generate a novel, renal tubule-specific RGC-32 knockout mouse model and systematically characterized its phenotype. Our findings demonstrate that renal tubule-specific RGC-32 deficiency does not impair normal growth or baseline renal function but alters the distribution of peripheral blood T lymphocyte subsets; whether this alteration contributes to renal immune regulation remains to be determined by future functional studies. More importantly, upon IRI, RGC-32 knockout in renal tubules leads to significantly aggravated renal dysfunction, elevated injury markers, and a possible association with enhanced chronic fibrosis.

## Simple Summary

RGC-32 participates in the repair process following renal injury. In this study, we established a renal tubule-specific RGC-32 knockout mouse model and systematically characterized the phenotypic features of this animal model. Renal ischemia–reperfusion injury (IRI) was induced, followed by quantitative assessment of the severity of renal injury to clarify the biological role of RGC-32 in renal repair following IRI.

## 1. Introduction

Acute kidney injury (AKI) and chronic kidney disease (CKD) are prevalent worldwide, imposing a substantial burden of morbidity, mortality, and healthcare costs across diverse socioeconomic settings. CKD is projected to become the fifth leading cause of death globally by 2040 [1,2], underscoring its growing public health impact. Tubular injury/Tubular cell death is a hallmark of AKI [3,4]. However, renal tubular epithelial cells exhibit a robust reparative capacity. Consequently, the repair process following renal tubular injury largely determines the prognosis and outcome of AKI, which can be classified into two distinct categories [5,6]. The first is adaptive repair, characterized by complete restoration of renal tubular structure and function. The second is maladaptive repair, characterized by excessive proliferation of tubular epithelial cells and extensive extracellular matrix synthesis, which ultimately progresses to CKD. Currently, the mechanisms that determine why some patients with AKI achieve adaptive repair and fully recover renal tubular function, while others undergo maladaptive or delayed repair and subsequently progress to CKD, remain not fully elucidated. Although the prevalence of AKI and CKD remains high and effective therapeutic targets remain scarce, significant progress has been made in recent years across the following major directions: G2/M phase cell cycle arrest [7], DNA damage [8], mitochondrial dysfunction [9], hypoxia-inducible factor signaling [10], dysregulated autophagy [11], and epigenetic alterations [12].

*RGC-32* (Response Gene to Complement 32), also termed *RGCC* (Regulator of Cell Cycle), is a gene mapped to human chromosome 13q14.11 [13]. It consists of five exons and four introns [14] and encodes a 137-amino-acid protein. First identified in rats by Horea Rus and colleagues in 1998, *RGC-32* was characterized as a complement activation-inducible gene [15]. Initially, RGC-32 was presumed to be exclusively triggered by complement activation and function as a cell cycle regulator. Nevertheless, accumulating evidence from subsequent studies has demonstrated that this gene participates in a wide spectrum of biological processes, including cell proliferation and differentiation, inflammatory responses, cell cycle regulation, immune modulation, as well as tumorigenesis [16].

RGC-32 is abundantly expressed in all tubular segments of normal renal tissues, including the proximal tubules, distal tubules, and collecting ducts, and is primarily localized to the cytoplasm and perinuclear region of renal tubular epithelial cells [17]. Moreover, RGC-32 is involved in cell cycle regulation as well as cell proliferation and differentiation. Therefore, to explore the functional role of RGC-32 in renal repair after acute ischemia–reperfusion injury (IRI), we established a renal tubular-specific RGC-32 knockout mouse model via the CRISPR-Cas9 system combined with the Cre/loxP recombination system. Genotyping and phenotypic verification were performed to confirm the successful construction of the animal model. Subsequently, a classic renal IRI-induced acute kidney injury model, was constructed in a renal tubular-specific RGC-32 knockout mouse model. To assess the degree of renal damage, we detected serum cystatin C (Cys-C) levels, tubulointerstitial injury score, quantified the mRNA expression of neutrophil gelatinase-associated lipocalin (NGAL) in renal tissues, and analyzed the protein expression of fibronectin (FN) by immunohistochemistry in both renal tubular-specific RGC-32 knockout mice (hereinafter referred to as conditional knockout (CKO) mice) and wild type (WT) control groups.

## 2. Materials and Methods

### 2.1. Generation of Renal Tubule-Specific RGC-32 Knockout Mouse Model

Ethics Statement: All animal experiments were approved by the Ethics Committee of Shanghai Children’s Hospital (Approval No. SHCH-IACUC-2021-XMSB-36) and were performed in accordance with the institutional guidelines for the care and use of laboratory animals.

**Figure 1:**
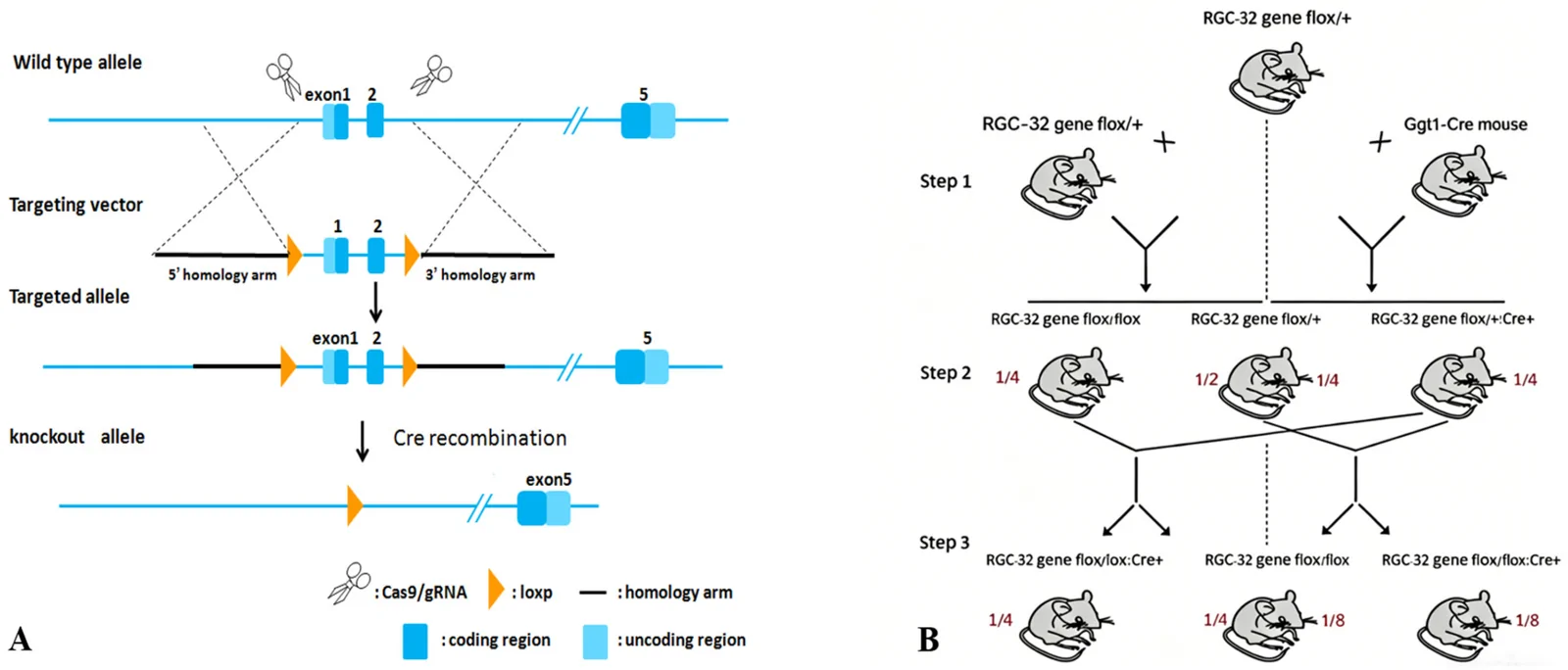
Schematic diagram of the mice generation strategy. (**A**) Schematic representation of the generation of tubule-specific RGC-32 knockout mice. Upon Cre-mediated recombination specifically in kidney tubule epithelial cells, exons 1–2 of the RGC-32 gene are excised. (**B**) Breeding strategy for generating experimental mice. Mice with the RGC-32 flox/+ genotype were crossed with Ggt1-Cre transgenic mice. Through the indicated breeding scheme, the experimental group RGC-32 flox/flox; Cre+ mice were obtained.

Mouse model generation: The RGC-32 conditional knockout mouse line was generated by Shanghai Model Organisms Center, Inc. (Shanghai, China) using CRISPR/Cas9-mediated homology-directed repair (HDR) at the zygote stage (strategy diagram shown in Figure 1A). Four sgRNAs targeting exons 1–2 of the RGC-32 gene (Ensembl: ENSMUSG00000022018; transcript ENSMUST00000022595.7) were designed and synthesized (sgRNA sequences are listed in Supplementary Material S1 Section S3.2). Cas9 mRNA and sgRNAs were obtained by in vitro transcription. Meanwhile, a homologous recombination donor vector was constructed using In-Fusion cloning, which contained a 3.0 kb 5′ homology arm, a 1.4 kb floxed region (including the target exons flanked by two loxP sites), and a 3.0 kb 3′ homology arm. The Cas9 mRNA, sgRNAs, and donor vector were co-microinjected into fertilized zygotes of C57BL/6J mice, and F0 mice were obtained via homologous recombination-mediated repair. PCR- and sequencing-positive F0 mice were then crossed with wild-type C57BL/6J mice to generate F1 floxed heterozygous mice with stable germline transmission. All PCR products from positive F1 mice were verified by Sanger sequencing. The sequence alignment results are presented in Supplementary Material S1 Section S5, confirming the correct integration of the 5′ and 3′ homology arms and the flanking loxP sites.

Subsequently, floxed mice were crossed with Ggt1-Cre transgenic mice (purchased from Cyagen Model Organisms Research Center Co., Ltd. (Suzhou, China)), which express Cre recombinase specifically in renal tubular epithelial cells. Through a multi-generation breeding strategy (Figure 1B), we ultimately obtained experimental mice (CKO mice) with renal tubular epithelial cell-specific knockout of the RGC-32 (genotype: RGC-32 flox/flox; Cre+). All animals were housed in the specific pathogen-free (SPF) facility at the National Center for Protein Science in Shanghai, China.

### 2.2. Genotyping and Validation of Knockout Efficiency in Tubule-Specific RGC-32 Knockout Mouse Model

#### 2.2.1. Genotyping

Genomic DNA was extracted from approximately 0.5–1 cm tail clips collected from 3- to 4-week-old offspring using a commercial kit (Ezup Column Animal Genomic DNA Purification Kit, Sangon Biotech, Shanghai, China; B518251). Genotyping was performed by polymerase chain reaction (PCR) with allele-specific primers (sequences are listed in Table 1). Primer pairs P1/P2 and P3/P4 were used to detect the floxed RGC-32 allele and the Cre transgene, respectively. Primer pair P5/P6 was designed to verify Cre-mediated excision of the floxed allele, where the forward primer was located upstream of the 5′ loxP site and the reverse primer downstream of the 3′ loxP site, generating a truncated product exclusively in the target tissue of CKO mice. PCR products were resolved by agarose gel electrophoresis.

#### 2.2.2. Verification of Knockout Efficiency

To confirm the effective knockout of the target gene in the intended tissue, kidneys were isolated from 6- to 8-week-old male CKO and WT mice for validation.

Quantitative real-time PCR (qRT-PCR): Total RNA was extracted from kidney using the RNA Extraction Kit (Beyotime, Shanghai, China; R0027) and reverse transcribed into cDNA. qRT-PCR was performed with gene-specific primers (sequences are listed in Table 2). The expression levels of the target gene were normalized to that of the internal control gene GAPDH. Each sample was analyzed in triplicate, and the mean value was used for subsequent analysis.

Western blot analysis: Protein samples were separated by SDS-PAGE and transferred onto PVDF membranes via wet electroblotting (300 mA, 30 min). The membranes were blocked and then incubated with primary antibodies targeting GAPDH (ET1601-4; 1:50,000; Hangzhou, China; HUABIO) or RGC-32 (sc-84223; 1:3000; Santa Cruz Biotechnology, Dallas, TX, USA), followed by incubation with horseradish peroxidase-conjugated secondary antibody (HA1001; 1:50,000–1:100,000; Hangzhou, China; HUABIO). Immunoreactive bands were visualized using the Pierce™ ECL Western Substrate Kit (Thermo Fisher Scientific, Rockford, IL, USA). PageRuler™ Prestained Protein Ladder (Thermo Scientific™, Waltham, MA, USA; 26616, 10–180 kDa) was used as a molecular weight marker. The gray value of each protein band was quantified using ImageJ2 software (v.2.14.0/1.54f). The gray value of the target protein was normalized to that of the internal reference protein GAPDH to calculate the relative protein expression level. Four independent biological replicates were performed for statistical analysis.

### 2.3. Phenotypic Observation and Analysis

#### 2.3.1. General Condition and Growth Monitoring

Body weights of mice in the CKO and WT groups were recorded weekly from 3 to 10 weeks of age to generate growth curves. In parallel, survival and fertility were monitored.

#### 2.3.2. Blood Analysis

Male mice (approximately 8 weeks old) were fasted for 6 h (with free access to water) before blood collection. Whole blood was obtained from the retro-orbital venous plexus.

Complete Blood Count (CBC): A portion of the blood sample was collected into an anticoagulant tube and analyzed using an automated hematology analyzer for a complete blood count (CBC).

Renal Function Assessment: Another portion was centrifuged to obtain serum. Serum creatinine (CREA) levels were measured using an automated biochemistry analyzer to assess renal function.

Flow Cytometric Analysis of T Cells: A further portion of anticoagulated blood was treated with red blood cell lysis buffer to obtain a mononuclear cell suspension. The cells were then sequentially subjected to Fc receptor blocking (BD 553141), live/dead cell discrimination staining (BD 564406), and staining with specific fluorescent antibodies against T cell surface markers CD3+ (BD 553061), CD4+ (BD 550954), and CD8+ (BD 566985). Stained cells were then acquired on a flow cytometer for data analysis, and data were analyzed using standard methods.

### 2.4. Establishment of the Mouse Model of Renal IRI and Specimen Collection

The renal IRI mouse model was established using the standard bilateral renal pedicle clamping method [18]. WT and CKO mice, aged 6–8 weeks and weighing 20–25 g, were used in this study.

Prior to surgery, the animals were fasted for 12 h and anesthetized via intraperitoneal injection of sodium pentobarbital. Following successful anesthesia, a midline abdominal incision was made to expose the peritoneal cavity. Both renal pedicles were carefully isolated and exposed. Micro-arterial clamps were then applied to occlude the bilateral renal pedicles.

Successful induction of ischemia was confirmed by a visible color change in the kidneys from bright red to dark red. The ischemic period lasted 45 min, during which body temperature was maintained and the tissues were kept moist. After 45 min, the clamps were removed. Successful reperfusion was verified by the rapid restoration of kidney color to bright red within seconds. The abdominal cavity was subsequently closed in layers, and the wound was disinfected. Postoperatively, the mice were returned to their cages for routine care. Sham-operated control mice underwent an identical surgical procedure without clamping.

Following model establishment, the mice (*n* = 3 per group per time point) were euthanized at predetermined time points post-reperfusion: 6 h, 24 h, 48 h, 72 h, 1 week, 2 weeks, 4 weeks, and 8 weeks. At each time point, whole blood samples were collected via the retro-orbital venous plexus. Subsequently, transcardiac perfusion with physiological saline was performed to flush out residual blood from the kidneys. Both kidneys were rapidly harvesteded and bisected along the coronal plane. One half of each kidney was immediately snap-frozen in liquid nitrogen and stored at −80 °C for subsequent analyses. The other half was fixed in 4% paraformaldehyde at 4 °C for 24–48 h, followed by paraffin embedding.

### 2.5. Assessment of Renal Injury Severity After Renal IRI

Serum cys-C measurement: Frozen serum aliquots preserved at −80 °C were thawed, and serum cys-C concentrations were quantified on a fully automated cystatin C analyzer (Beckman Coulter Shanghai Jingyuan Medical Instrument Co., Ltd. Shanghai, China).

Histological assessment of tubulointerstitial injury score: Kidney tissues were fixed and embedded in paraffin, sectioned at 3–4 μm thickness, and stained with hematoxylin and eosin (H&E) for histopathological examination. Tubulointerstitial injury was evaluated semi-quantitatively according to the method described by Sayhan et al. [19]. Under light microscopy at ×200 magnification, 10 randomly selected non-overlapping tubulointerstitial fields were examined per section. The following four parameters were assessed: (1) tubular atrophy; (2) tubular degeneration and necrosis; (3) interstitial inflammatory cell infiltration; and (4) interstitial fibrosis. Histopathological features evaluated included tubular swelling, degeneration, necrosis, atrophy, loss of brush border, interstitial edema, and inflammatory cell infiltration. Each parameter was graded on a scale of 0 to 4 based on the percentage area of involvement: 0 = no injury (0%); 1 = mild injury (<25%); 2 = moderate injury (25–50%); 3 = severe injury (51–75%); and 4 = very severe injury (>75%). All evaluations were performed by two independent pathologists blinded to the experimental groups. The total tubulointerstitial injury score was calculated as the sum of the four sub-scores.

qRT-PCR for renal tissue NGAL mRNA expression: The procedure was performed as described in Section 2.2, with specific primers (sequences are listed in Table 2).

Immunohistochemical staining and quantitative analysis of FN: Paraffin-embedded renal tissue sections were subjected to FN immunohistochemistry. Briefly, slides were deparaffinized in xylene and rehydrated via a descending ethanol gradient. Antigen retrieval was achieved by heating sections in citrate buffer (pH = 6.0). Endogenous peroxidase activity was quenched using 3% hydrogen peroxide solution. Subsequently, sections were incubated overnight at 4 °C with primary anti-fibronectin antibody (Abcam, Cambridge, UK; ab2413), followed by incubation with horseradish peroxidase (HRP)-conjugated goat anti-rabbit IgG secondary antibody (Jackson ImmunoResearch Laboratories, West Grove, PA, USA; 111-035-003). Immunoreactive signals were developed with 3,3′-diaminobenzidine (DAB) chromogen, and cell nuclei were counterstained with hematoxylin.

For semi-quantitative evaluation, 10 random non-overlapping visual fields per section were photographed under 200× magnification. ImageJ software was applied to measure FN-positive stained areas and total tissue area within each field. The percentage of FN-positive area was computed for each field, and the mean value of the 10 fields was adopted for subsequent statistical comparisons. A higher proportion of FN-positive staining reflects aggravated renal interstitial fibrosis.

### 2.6. Statistical Analysis

Data are presented as mean ± standard deviation (mean ± SD). Statistical analyses were performed using GraphPad Prism software (version 9.0; GraphPad Software, San Diego, CA, USA). Between-group comparisons were analyzed using an independent-samples Student’s *t*-test when the data met the assumptions of normality and homogeneity of variances; otherwise, the Mann–Whitney U test was applied. Given the multiple observation time points and the relatively small sample size after ischemia–reperfusion injury, a two-way analysis of variance (ANOVA) was performed to evaluate the main effects of genotype and time, as well as their interaction across the entire time course. Following the two-way ANOVA, pairwise comparisons between the WT and CKO groups at each individual time point were conducted using unpaired Student’s *t*-tests, and the resulting *p* values were adjusted using the Benjamini–Hochberg false discovery rate (FDR) correction to account for multiple testing. Sample size was *n* = 3 per group at each time point. A *p* value < 0.05 (adjusted *q* < 0.05 after FDR correction) was considered statistically significant.

## 3. Results

### 3.1. Successful Generation and Validation of Renal Tubule-Specific RGC-32 Knockout Mouse Model

#### 3.1.1. Genotyping Results

**Figure 2:**
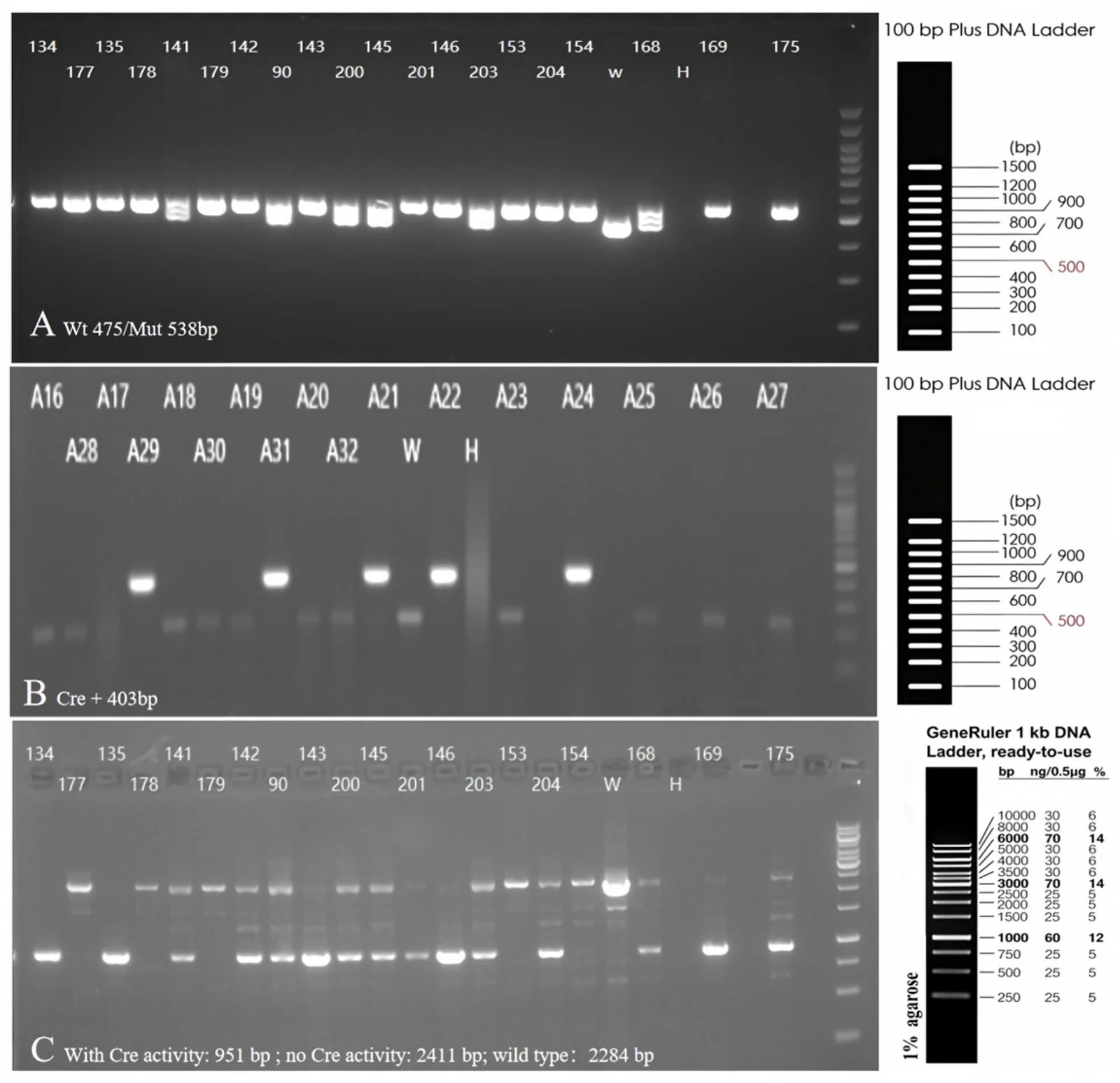
Genotyping PCR results of mice. W: wild type control; H: water negative control. (**A**) PCR genotyping of the floxed RGC-32 allele. The wild type allele yields a 475 bp product, the homozygous floxed allele (flox/flox) yields a 538 bp product, and the heterozygous floxed allele (flox/+) yields both 475 bp and 538 bp products. (**B**) Detection of the Cre transgene. A 403 bp specific band is amplified in Cre-positive mice. (**C**) Genotyping for Cre-mediated deletion of the floxed allele: Cre-mediated recombination successfully deleted the floxed RGC-32 allele, yielding a 951 bp product, incomplete recombination produced both 951 bp and 2411 bp products, whereas the absence of recombination produced only the 2411 bp product.

Genotyping of the RGC-32 Floxed Allele: The WT allele generated a 475 bp PCR product, whereas the homozygous floxed (flox/flox) allele produced a 538 bp product. Heterozygous floxed (flox/+) allele exhibited two distinct bands corresponding to 475 bp and 538 bp. After systematic genotyping screening, RGC-32 flox/flox mice were successfully acquired for subsequent mating and animal breeding experiments (Figure 2A).

Genotyping for Cre Recombinase: To establish a renal tubule-specific knockout mouse model, RGC-32 flox/flox mice were crossbred with Ggt1-Cre transgenic mice, which exhibit specific Cre recombinase expression in renal tubular epithelial cells. PCR-based genotyping confirmed the presence of the Cre transgene in offspring, with a specific amplicon of 403 bp detected in Cre-positive mice (Figure 2B).

Genotyping for Cre-mediated deletion of the floxed allele: Cre had indeed mediated recombination, and the RGC-32 gene had been conditionally deleted, as indicated by an amplified product of 951 bp. In the case of incomplete recombination, both the 951 bp and 2411 bp amplicons were detected. When no Cre-mediated recombination had occurred, only the 2411 bp product was observed (Figure 2C).

Mice that tested positive for all three pairs of primers—P1/P2 (a 538 bp band), P3/P4 (a 403 bp band), and P5/P6 (a 951 bp band)—were confirmed as our experimental mice, representing a stable renal tubular epithelial cell-specific RGC-32 knockout mouse model. These genotyped experimental mice were housed together with age-matched (3–4 weeks old) wild-type male mice. Mice of other genotypes, such as the flox/flox; Cre-negative and Cre-only controls, were identified by genotyping and then euthanized.

#### 3.1.2. Validation of Knockout Efficiency

**Figure 3:**
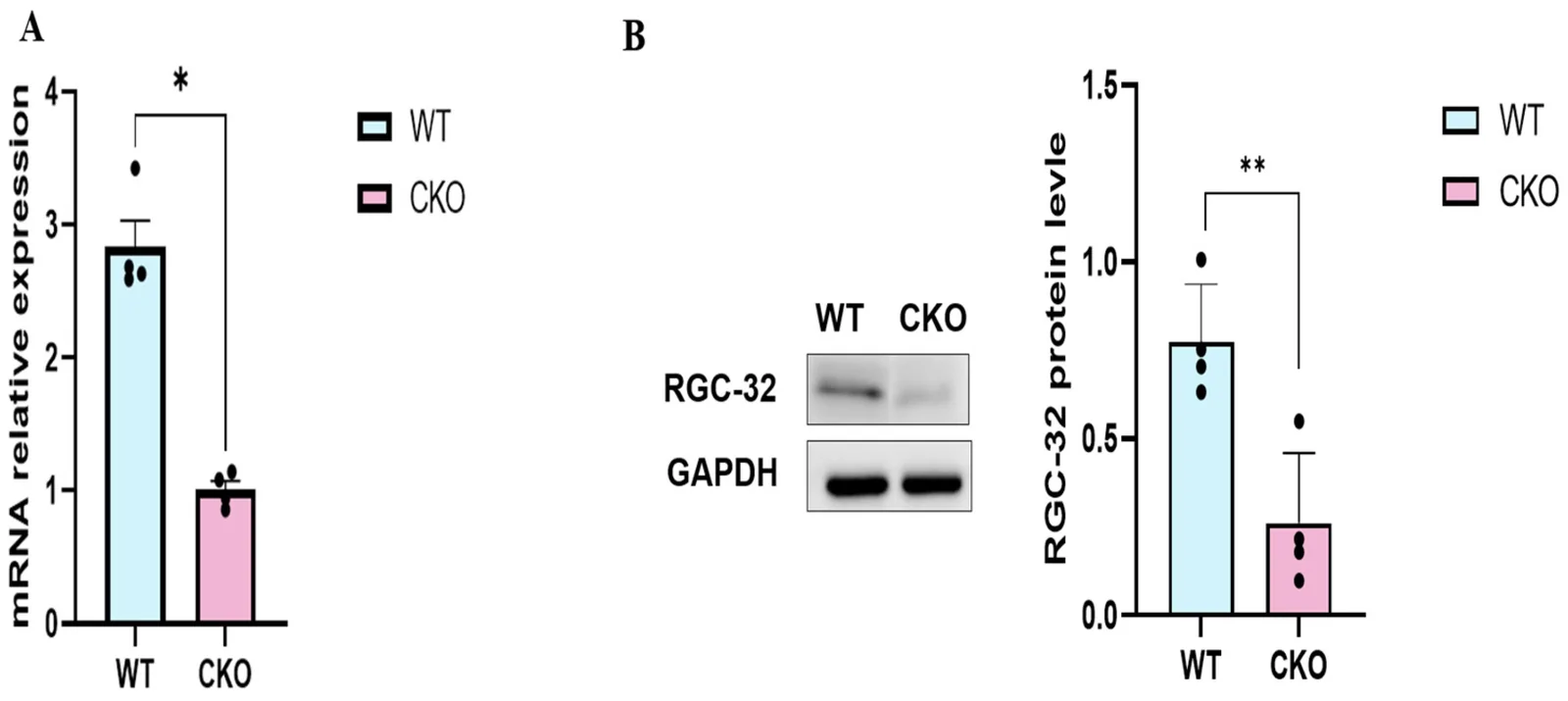
Validation of RGC-32 knockout efficiency in the kidney. (**A**) mRNA expression levels of RGC-32 in the kidney tissues of WT and CKO mice (*n* = 4 per group). Relative mRNA expression was normalized to an internal reference *GAPDH* gene and was shown as the mean ± SD. RGC-32 mRNA expression was significantly reduced in CKO mice compared with WT controls (* *p* < 0.05). (**B**) Protein expression levels of RGC-32 in the kidney tissues of WT and CKO mice (*n* = 4). GAPDH served as the loading control. Densitometric values were normalized to GAPDH and are expressed as the mean ± SD relative to WT. RGC-32 protein expression was significantly reduced in CKO mice compared with WT controls (** *p* < 0.01) (The original Western blot images were supplied in Supplementary Material S2).

qRT-PCR: qRT-PCR analysis revealed that RGC-32 mRNA levels in the kidneys of CKO mice were significantly lower than those in control mice (*p* < 0.05; Figure 3A).

Western blot: Western blotting further confirmed a marked reduction in RGC-32 protein expression in the renal tissues of CKO mice compared with controls (*p* < 0.01; Figure 3B).

### 3.2. Phenotypic Observation and Analysis Result

#### 3.2.1. General Condition and Growth Monitoring Result

**Figure 4:**
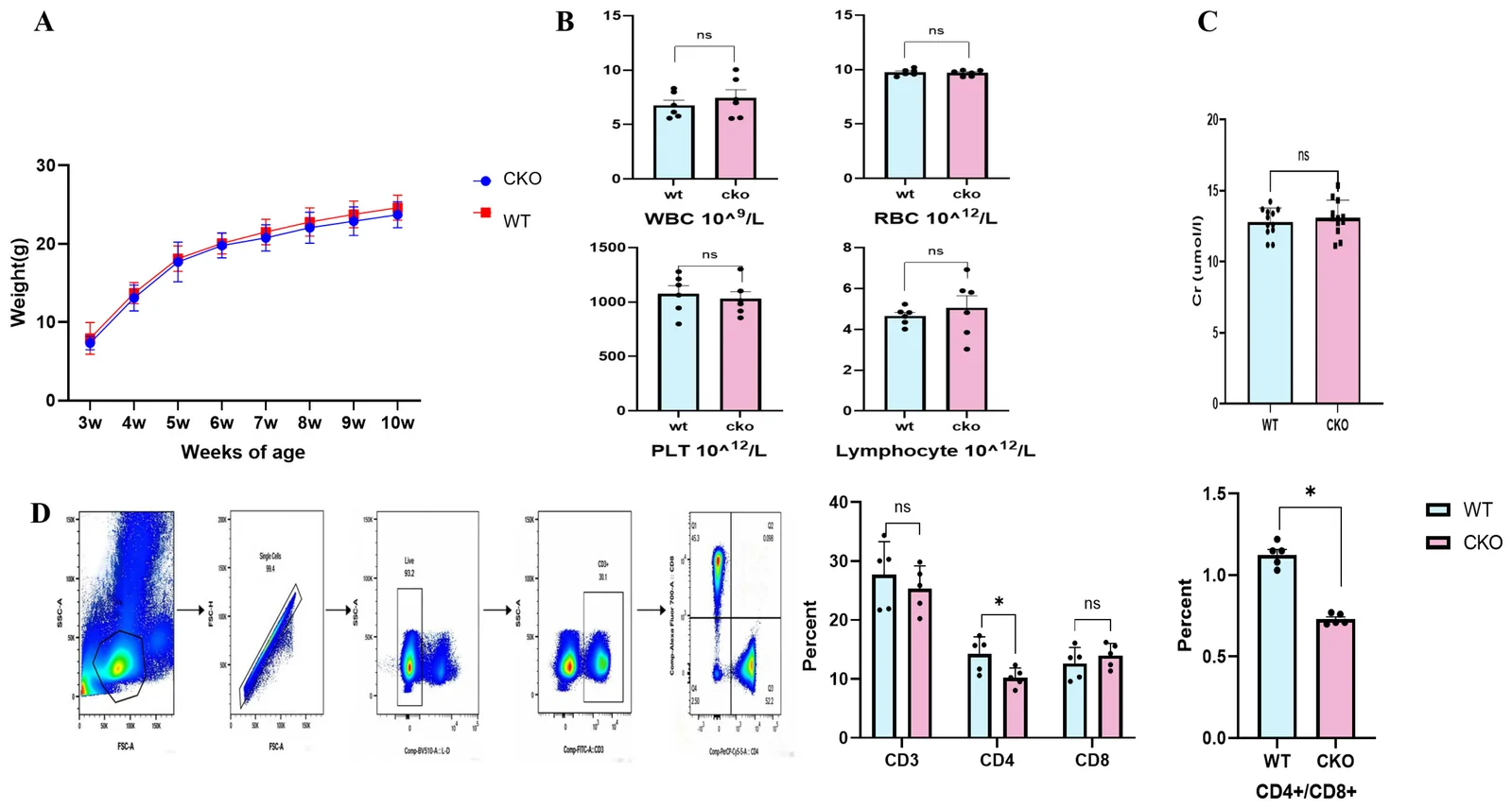
Phenotypic characterization of WT and CKO mice. (**A**) Body weight of WT and CKO mice from 3 to 10 weeks of age (*n* = 8). No significant difference in body weight was observed between the two groups throughout the observation period (*p* > 0.05). (**B**) Complete blood count parameters of WT and CKO mice (*n* = 6). No significant differences in white blood cell (WBC), red blood cell (RBC), platelet (PLT), and lymphocyte counts were observed between the two groups (*p* > 0.05). (**C**) Serum creatinine levels in WT and CKO mice (*n* = 12). No significant difference in serum creatinine was detected between the two genotypes (*p* > 0.05). (**D**) Peripheral blood T lymphocyte subsets in WT and CKO mice (*n* = 5). Compared with WT controls, CKO mice showed a significant reduction in both the percentage of CD4+ T cells and the CD4+/CD8+ ratio (* *p* < 0.05). In contrast, the percentages of CD3+ and CD8+ T cells were comparable between the two groups, with no significant differences (*p* > 0.05). ns: not statistically significant.

CKO mice displayed normal reproductive capacity and survival status, with no spontaneous tumors, abnormal mortality, or other adverse events observed. Although body weight increased gradually with age, no significant differences were detected between WT and CKO mice at any time point (*p* > 0.05; Figure 4A). Collectively, these results demonstrate that tubule-specific ablation of RGC-32 exerts no adverse effects on the normal growth and development of mice.

#### 3.2.2. Blood Analysis Result

Peripheral blood analysis revealed no significant differences in white blood cell count, red blood cell count, platelet count, or lymphocyte count between adult WT and CKO mice (*p* > 0.05; Figure 4B). These findings indicate that tubule-specific deletion of RGC-32 does not adversely affect the hematopoietic system.

Similarly, serum creatinine levels were comparable between the two genotypes (*p* > 0.05; Figure 4C), suggesting that renal tubule-specific RGC-32 ablation does not impair basal renal function in mice.

Given that *RGC-32* is a complement-responsive gene with established roles in immune modulation, we next evaluated peripheral T-lymphocyte subpopulations. Flow cytometric analysis demonstrated a significant reduction in both the percentage of circulating CD4+ T cells and the CD4+/CD8+ ratio in CKO mice relative to WT controls (*p* < 0.05; Figure 4D). In contrast, the proportions change in CD3+ and CD8+ T cells remained showed no significant differences between the two groups, indicating a selective effect on the CD4+ compartment.

### 3.3. Assessment of Renal Injury Following Renal IRI

To evaluate the severity of renal injury in CKO and WT mice following renal IRI, multiple renal damage-related indicators were systematically detected and compared between the two groups.

**Figure 5:**
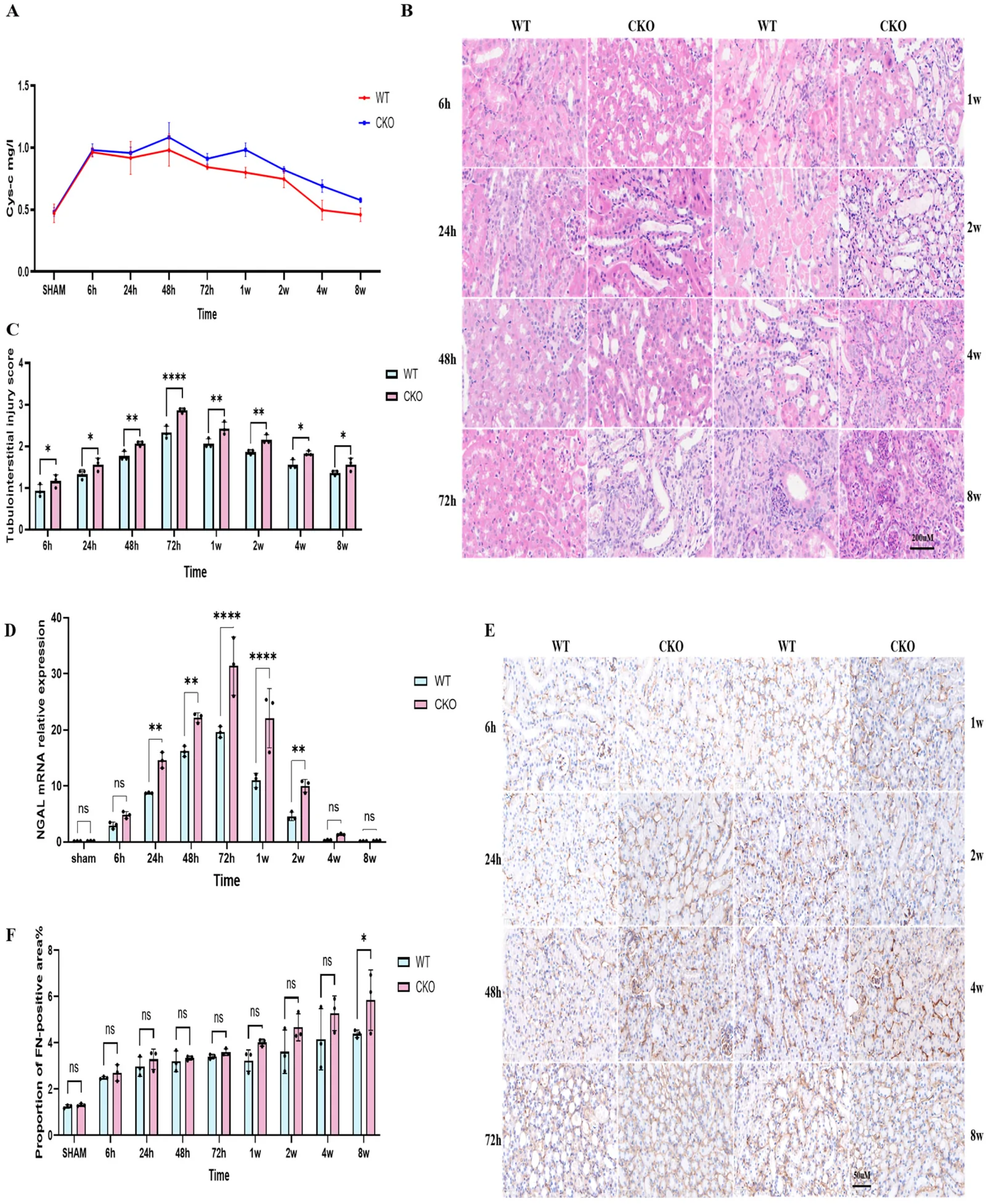
Assessment of renal injury after IRI. (**A**) Serum Cys-C levels in WT and CKO mice at the indicated time points after IRI (*n* = 3 per group per time point). Data are shown as mean ± SD. (BH-FDR-corrected unpaired *t* test); CKO mice showed significantly higher Cys-C than WT at 1 week (*q* = 0.012) and 4 weeks (*q* = 0.012). The difference at 8 weeks was nominally significant by an uncorrected *t* test (*p* = 0.048) but did not survive FDR correction (*q* = 0.143). (**B**) Representative images of HE-stained kidney sections from WT and CKO mice after IRI. 200×, Scale bar, 200 µm. (**C**) Tubulointerstitial injury score (*n* = 3 per group per time point) were shown as mean ± SD. * *q* < 0.05 (BH-FDR-corrected unpaired *t* test); CKO mice showed significantly higher injury scores than WT at all time points tested (6 h to 8 weeks; all *q* < 0.05). The difference was greatest at 72 h after injury (**** *q* < 0.0001). (**D**) Renal NGAL mRNA expression in WT and CKO kidneys after IRI, normalized to GAPDH gene (*n* = 3 per group per time point). Data are shown as mean ± SD. * *q* < 0.05 (BH-FDR-corrected unpaired *t* test). NGAL mRNA was markedly higher in CKO than in WT mice from 24 h to 2 weeks after injury, with the largest differences at 72 h (**** *q* < 0.0001) and 1 week (**** *q* < 0.0001), and remained significant at 24 h (*q* = 0.0012), 48 h (*q* = 0.0011), and 2 weeks (*q* = 0.0019). No significant difference was detected at sham, 6 h, 4 weeks, or 8 weeks (all *q* > 0.05). (**E**) Representative FN immunohistochemical staining of WT and CKO kidney sections at the indicated time points after IRI. 200×, Scale bar, 50 µm. (**F**) Quantitative analysis of FN immunostaining in WT and CKO kidneys after IRI, expressed as a percentage of positively stained area (*n* = 3 per group per time point). Data are shown as mean ± SD. * *q* < 0.05 (BH-FDR-corrected unpaired *t* test). FN immunostaining was significantly higher in CKO than in WT only at 8 weeks (*q* = 0.0408); the differences at 2 weeks (*p* = 0.0339) and 4 weeks (*p* = 0.0251) were nominally significant but did not survive FDR correction (*q* = 0.1018 for both). * *q* < 0.05, ** *q* < 0.01, **** *q* < 0.0001, ns: not statistically significant.

Serum Cys-C is widely used for the early diagnosis of renal impairment. Two-way ANOVA revealed a significant main effect of time (F_8,36_ = 52.95, *p* < 0.0001) and of genotype (F_1,36_ = 22.18, *p* < 0.0001), with no significant genotype × time interaction (F_8,36_ = 1.368, *p* = 0.244), indicating that CKO mice showed consistently higher CysC levels across the time course. Point-by-point unpaired *t* tests with Benjamini–Hochberg FDR correction confirmed that CKO mice exhibited significantly higher CysC levels than WT controls at 1 week (*q* = 0.012) and 4 weeks (*q* = 0.012). Although the difference at 8 weeks reached nominal significance by an uncorrected test (*p* = 0.048), it did not survive FDR correction (*q* = 0.143), and the remaining time points (SHAM, 6 h, 24 h, 48 h, 72 h, and 2 weeks) were not significantly different between genotypes (Figure 5A *n* = 3 per time point per group). These data indicate that genetic ablation predominantly aggravated renal dysfunction at the intermediate time points after injury.

The tubulointerstitial injury score is a semi-quantitative tool for objectively assessing tubular atrophy and interstitial fibrosis (Figure 5B), and is widely used to reflect renal functional impairment and predict renal disease prognosis. Two-way ANOVA revealed a significant main effect of time (F_7,32_ = 104.9, *p* < 0.0001) and of genotype (F_1,32_ = 79.54, *p* < 0.0001), with no significant genotype × time interaction (F_7,32_ = 1.209, *p* = 0.326), indicating that CKO mice displayed consistently higher injury scores throughout the time course. Point-by-point unpaired *t* tests with Benjamini–Hochberg FDR correction confirmed that CKO mice had significantly higher tubular injury scores than WT controls at every time point examined, from 6 h to 8 weeks (all *q* < 0.05; nominal *p* values from 0.0039 to 0.046, all of which remained significant after correction; Figure 5C *n* = 3 per time point per group). The difference was greatest at 72 h after injury (*q* < 0.0001). These findings indicate that genetic ablation consistently aggravated tubulointerstitial injury at all stages from the acute (hours) to the chronic (weeks) phase after IRI.

Kidney NGAL mRNA expression is a well-recognized high-sensitivity and high-specificity biomarker for AKI. Two-way ANOVA revealed significant main effects of time (F_8,36_ = 146.5, *p* < 0.0001) and of genotype (F_1,36_ = 88.06, *p* < 0.0001), as well as a significant genotype × time interaction (F_8,36_ = 8.433, *p* < 0.0001), indicating that the effect of genotype was time-dependent rather than uniform across the time course. Point-by-point unpaired *t* tests with Benjamini–Hochberg FDR correction showed that NGAL expression was markedly higher in CKO than in WT mice from 24 h to 2 weeks after injury, with the largest difference at 72 h (*q* < 0.0001) and 1 week (*q* < 0.0001); the difference remained significant at 24 h (*q* = 0.0012), 48 h (*q* = 0.0011), and 2 weeks (*q* = 0.0019) (Figure 5D *n* = 3 per time point per group). By contrast, no significant difference was detected at sham, 6 h, 4 weeks, or 8 weeks (all *q* > 0.05). These data indicate that genetic ablation augmented NGAL induction specifically during the acute injury phase (24 h–2 weeks), consistent with aggravated tubular injury in the early stage after ischemia–reperfusion.

Renal FN accumulation is a key pathological feature that drives the progression of acute kidney injury to CKD (Figure 5E). Immunohistochemical staining showed that two-way ANOVA revealed a significant main effect of time (F_8,36_ = 22.60, *p* < 0.0001) and of genotype (F_1,36_ = 13.74, *p* = 0.0007), with no significant genotype × time interaction (F_8,36_ = 1.160, *p* = 0.3495), indicating that FN protein levels tended to be uniformly higher in CKO mice across the time course. Point-by-point unpaired *t* tests with Benjamini–Hochberg FDR correction, however, showed that the elevation reached statistical significance only at 8 weeks after injury (*q* = 0.0408). The differences at 2 and 4 weeks were nominally significant by uncorrected tests (*p* = 0.0339 and *p* = 0.0251, respectively) but did not remain significant after FDR correction (*q* = 0.1018 for both), and no significant difference was detected at the remaining time points (SHAM through 1 week; all *q* > 0.25) (Figure 5F *n* = 3 per time point per group). These data indicate that FN accumulation was mildly but persistently increased in CKO kidneys and became statistically discernible predominantly at the late (8-week) time point, consistent with a pro-fibrotic tendency in the chronic phase after IRI.

## 4. Discussion

The human *RGC-32* gene encodes a 137-amino-acid protein that shares 92% sequence identity with its rat and mouse orthologs. RGC-32 is highly expressed in the kidney, liver, placenta, pancreas, skeletal muscle, and aortic endothelial cells; however, its expression is weak in the brain and undetectable in the lung [16]. As a key complement-responsive gene, it plays pleiotropic roles in various biological processes, including cell cycle regulation, proliferation, differentiation, inflammatory responses, immune modulation, and tumorigenesis [20].

To precisely evaluate the role of RGC-32 in renal IRI, we generated a renal tubule-specific RGC-32 knockout mouse model using CRISPR-Cas9 and Cre/loxP technology and characterized its phenotypic features. CKO mice showed normal breeding, postnatal survival, and no spontaneous tumors or excessive mortality, and body weight tracking from 3 to 10 weeks revealed no differences between CKO and WT mice, indicating that conditional RGC-32 ablation does not affect growth. But Global RGC-32 knockout causes systemic and lifelong loss of the gene across all tissues and cell types [21]; such mice reportedly exhibit −27% lower birth weight than WT littermates, a difference persisting until 4 weeks of age but disappearing by 5 weeks owing to catch-up growth. This phenotype has been attributed to reduced expression of placental angiogenesis-related genes (*VEGFR2* and *PlGF*), leading to impaired placental angiogenesis, intrauterine growth restriction, and occasional embryonic lethality. Accordingly, to circumvent the systemic, developmental, and paracrine confounding effects that accompany global deletion, we employed a renal tubule-specific RGC-32 conditional knockout mouse model. This approach restricts gene ablation to the target cell type, as such, the CKO model more accurately reflects the role of RGC-32 in the IRI.

RGC-32 has been reported to modulate the functional activities of multiple immune cells. Previous studies have demonstrated that RGC-32 deficiency leads to dysregulation of T-cell subset proportions [22] and compromises T-cell immune function [23]. In the present study, we characterized peripheral T-cell subsets in CKO and WT mice by flow cytometry. Compared with WT mice, CKO mice exhibited significantly decreased proportions of circulating CD4+ T cells and a reduced CD4+/CD8+ ratio, with statistically significant differences observed between the two groups. These results indicate that renal tubule-specific RGC-32 deletion is associated with an altered distribution of peripheral T-lymphocyte subsets in this model; whether this alteration contributes to renal immune regulation remains to be determined by future functional studies. Furthermore, previous mechanistic studies have shown that RGC-32 modulates the proliferation and activation of different T-cell subpopulations via the NF-κB and PI3K/Akt signaling pathways [24]; however, these pathways were not examined in the present study, and whether they underlie the subset changes observed here requires further investigation.

RGC-32 exhibits pleiotropic functions across diverse pathological contexts and appears to act in a cell type- and injury context-dependent manner. In pulmonary fibrosis, high RGC-32 expression has been proposed to exert a protective effect [25]. Conversely, in multiple malignancies—including ovarian, colon, breast, and prostate cancers—elevated RGC-32 levels correlate with poor patient prognosis [22]. In renal diseases, accumulating evidence confirms that RGC-32 participates in renal tubular injury repair and is tightly linked to renal interstitial fibrosis [26]. However, its precise role in renal fibrosis—whether pro-fibrotic, anti-fibrotic, or mediated by a context-dependent bidirectional mechanism—remains controversial. In vitro studies using rat renal tubular epithelial cells demonstrated that siRNA-mediated RGC-32 knockdown triggers G2/M phase cell cycle arrest and upregulates the expression of core pro-fibrotic markers, including α-SMA and FN [27], implying a protective role of RGC-32 against renal fibrosis. Paradoxically, in a unilateral ureteral obstruction (UUO) mouse model, global RGC-32 knockout markedly alleviated UUO-induced renal structural injury, α-SMA expression, and collagen deposition, supporting a pro-fibrotic function of RGC-32 [28].

In our study, renal injury was comprehensively assessed across multiple complementary dimensions. Although serum creatinine remains the most widely used biomarker of kidney injury in humans, it is an insensitive and delayed indicator of acute kidney injury, a drawback that is particularly pronounced in rodent models. Indeed, previous studies have shown that serum creatinine levels may fail to rise appreciably in mice despite marked tubular injury [29]. We therefore employed the more sensitive endogenous biomarker Cys-C. Cys-C is a non-glycosylated protein produced at a constant rate by all nucleated cells, and its plasma concentration is almost exclusively determined by the glomerular filtration rate [30]. In the present study, serum Cys-C levels were significantly elevated in CKO mice, mainly at 1 and 4 weeks after IRI, indicating that functional deterioration became evident during the subacute phase. Histological evaluation (tubulointerstitial injury score) objectively assesses tubular atrophy and interstitial fibrosis and is widely used to reflect renal functional impairment and to predict renal disease prognosis. Our results demonstrated that morphological tubular injury was markedly aggravated in CKO kidneys, with significantly higher injury scores than WT controls at every time point examined from 6 h to 8 weeks. NGAL is a small glycoprotein predominantly expressed in renal tubular epithelial cells. Under physiological conditions, NGAL levels remain low in both urine and plasma; however, they rise rapidly after AKI, with abnormal levels detectable as early as 2 h post-injury, underscoring its utility as a novel biomarker with high sensitivity and specificity [31]. In our study, renal NGAL mRNA was markedly elevated in CKO kidneys at early-to-intermediate time points (24 h to 2 weeks). Renal interstitial fibrosis is a hallmark of CKD [32]. In our study, FN immunostaining was significantly higher in CKO kidneys at 8 weeks, indicating a shift toward progressive fibrosis. Taken together, these complementary markers demonstrate that tubule-specific *RGC-32* knockout aggravated both acute tubular injury and a possible association with enhanced chronic fibrosis after IRI. These results support a protective, a pro-fibrotic tendency of RGC-32 in the renal tubular epithelium.

Notably, this phenotype contrasts with the previous findings in the UUO model. UUO is a chronic, predominantly obstructive model that develops over days to weeks, during which sustained mechanical obstruction drives progressive tubulointerstitial fibrosis, tubular cell apoptosis, and interstitial inflammation, largely recapitulating the fibrotic end stage of CKD [33]. By contrast, IRI is an acute injury model characterized by initial ischemia followed by reperfusion-triggered oxidative stress, acute tubular necrosis, and a robust innate immune response. These two pathogenic cascades differ substantially in their time course, predominant cell death pathway, inflammatory microenvironment, and the type of infiltrating immune cells. This striking contrast highlights the functional complexity of RGC-32, which may elicit distinct, even opposing, injury responses in different contexts, potentially arising from its cell type-specific subcellular localization and differential responses to distinct pathological stimuli. Collectively, the necessity of cell-type-specific loss-of-function studies to elucidate the context-dependent roles of RGC-32 is highlighted.

Several limitations of this study should be acknowledged. First, because the time-course design included numerous observation time points (SHAM, 6 h, 24 h, 48 h, 72 h, 1 week, 2 weeks, 4 weeks, and 8 weeks), the sample size per group per time point (*n* = 3) was relatively small, which may have limited the statistical power to detect subtle differences at certain time points, particularly where the between-genotype differences reached only nominal significance. We therefore regard these results as informative preliminary findings, and we note that, despite this limitation, the consistency of the observed trends across the time course supports the overall conclusions of this study. Second, as for fibrosis, only FN was examined, which may not fully capture the fibrotic process. We therefore interpret our findings as evidence of a fibrotic tendency, and acknowledge that additional fibrosis markers would help to further consolidate this conclusion. Third, our analyses focused on RGC-32 protein- and mRNA-level readouts of injury and fibrosis; the specific upstream signaling pathways through which RGC-32 exerts these effects in tubular epithelial cells were not comprehensively dissected. Future studies with larger cohorts, a broader panel of fibrosis markers, and mechanistic dissection of the underlying pathways will help to confirm and extend our conclusions.

## 5. Conclusions

In conclusion, we established a tubule-specific RGC-32 conditional knockout mouse model and demonstrated that tubule-specific deletion of RGC-32 aggravated acute tubular injury and was associated with an increased fibrotic tendency after IRI, as reflected by elevated serum cystatin C, increased tubular injury scores, upregulated renal NGAL expression, and enhanced FN deposition. Notably, this aggravated phenotype contrasts with the alleviated injury observed in the UUO model, highlighting the pleiotropic, context-dependent nature of RGC-32 function. Our findings therefore underscore that the net effect of RGC-32 must be evaluated in light of the combined influence of the specific cell subpopulation, the signaling pathways engaged, and the pathological stimulus.

## Figures and Tables

**Table 1:** Primers used for genotyping tubule-specific RGC-32 knockout mice.

Primer	Sequence
P1-F	ACCCCCTTCTACCATCCTC
P2-R	TCTTTGCGGGCATCAGAGTT
P3-F	CAGCCTGCTCTAACGGTTTC
P4-R	CAGGTTCTTGCGAACCTCAT
P5-F	ACCCCCTTCTACCATCCTCC
P6-R	TTGTGCTCACTCGTGTCCTC

**Table 2:** Primer information for qRT-PCR.

Primer	Sequence
GAPDH-F	GGGTGTGAACCACGAGAAAT
GAPDH-R	CCTTCCACAATGCCAAAGTT
RGC-32-F	GAGCGCCACTTCCACTATGAG
RGC-32-R	TGAGTCTGCACTCTCCGAGTC
NGAL-F1	CAGTGCCGCCGATTACTACTT
NGAL-R1	AGGAGGCTAATAGTTTGTCGGAT

## Data Availability

The datasets generated during the current study are available from the corresponding author on reasonable request.

## Supplementary Materials

The following supporting information can be downloaded at: https://www.mdpi.com/article/10.3390/biology15171535/s1, Supplementary Material S1-Construction of a conditional gene knockout mouse model for RGC-32 (RGCC); Supplementary Material S2-Western blot.

## Abbreviations

The following abbreviations are used in this manuscript:

AKI	Acute kidney injury
CKD	Chronic kidney disease
Cys-C	Cystatin C
CKO	Conditional knockout
CBC	Complete Blood Count
CREA	Creatinine
FN	Fibronectin
IRI	Ischemia–reperfusion injury
NGAL	Neutrophil gelatinase-associated lipocalin
PCR	Polymerase chain reaction
qRT-PCR	Quantitative real-time PCR
RGCC	Regulator of Cell Cycle
RGC-32	Response Gene to Complement 32
UUO	Unilateral ureteral obstruction
WT	Wild type
