# Supplementary material for: Tubule-Specific RGC-32 Knockout Exhibits Direct and Progressive Aggravating Activity Against Renal Function in an Ischemia–Reperfusion Mouse Model
Source: Biology (Basel). 2026 Sep 4;15(17):1535. doi: 10.3390/biology15171535 (PMC13565509; doi:10.3390/biology15171535)
Supplement: Supplementary file 1 [file biology-15-01535-s001.zip › Supplementary Material S1- Construction of a conditional gene knockout mouse model for RGC-32 (RGCC).pdf]

Construction of a conditional gene knockout mouse model for RGC-32 (RGCC)

S1. Basic Information

Target gene name (Ensembl ID): Rgcc (ENSMUSG00000022018)

Reference gene Ensembl URL link:

http://asia.ensembl.org/Mus_musculus/Gene/Summary?db=core;

g=ENSMUSG00000022018; r=14:79288756-79301645; t=ENSMUST00000022595

The transcript targeted by the protocol (Ensembl ID): Rgcc-201 (ENSMUST00000022595.7)
Exons targeted by Flox: exon 1–2

S2. Abstract

This study employs the principle of homologous recombination, utilizing fertilized egg homologous recombination to perform flox modification on the Rgcc gene. The procedure is summarized as follows: Cas9 mRNA and gRNA were synthesized via in vitro transcription; a homologous recombination vector (donor vector) was constructed using In-Fusion cloning, comprising a 3.0 kb 5′ homologous arm, a 1.4 kb flox region, and a 3.0 kb 3′ homologous arm. The Cas9 mRNA, gRNA, and donor vector were microinjected into fertilized eggs of C57BL/6J mice to obtain F0-generation mice. Positive F0-generation mice were identified by PCR amplification and sequencing, and then crossed with C57BL/6J mice to produce six positive F1-generation mice.

S3. design strategy

S3.1. Schematic Diagram of Design Strategy:

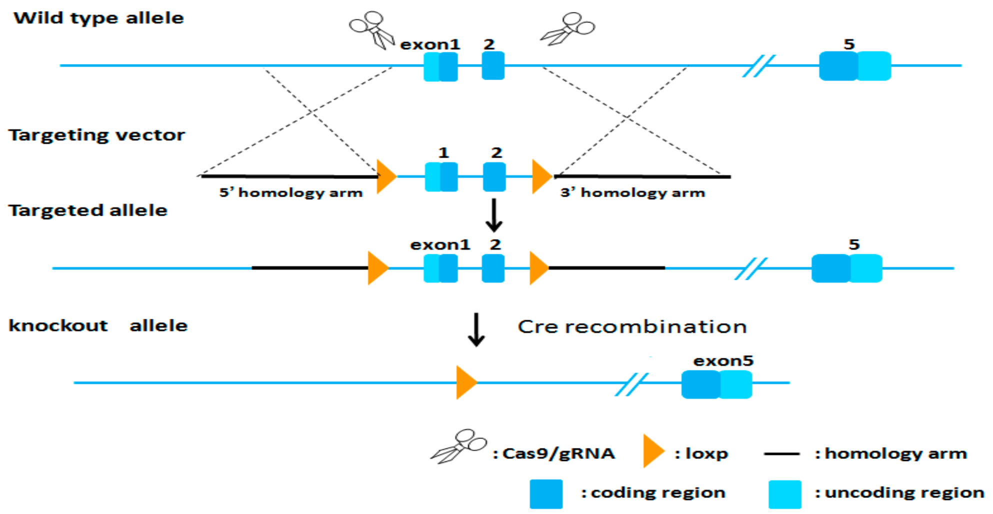

Figure S1: Schematic diagram of mouse construction strategy

S3.2. Sequence Information of gRNAs

Table S1: Sequence Information of gRNAs

**gRNAs**	**Sequence (5′-3′)**
**gRNA1**	TTACCCTGTTTCCCGCCTTC TGG
**gRNA2**	CTCAAGGAGGCTCAGCCAGA AGG
**gRNA3**	GGGGAGAGAGGTACGAGGGT GGG
**gRNA4**	TCTTGGGGAGAGAGGTACGA GGG

S4. Construction of homologous recombination plasmids

S4.1 Homologous Recombination Plasmid Atlas

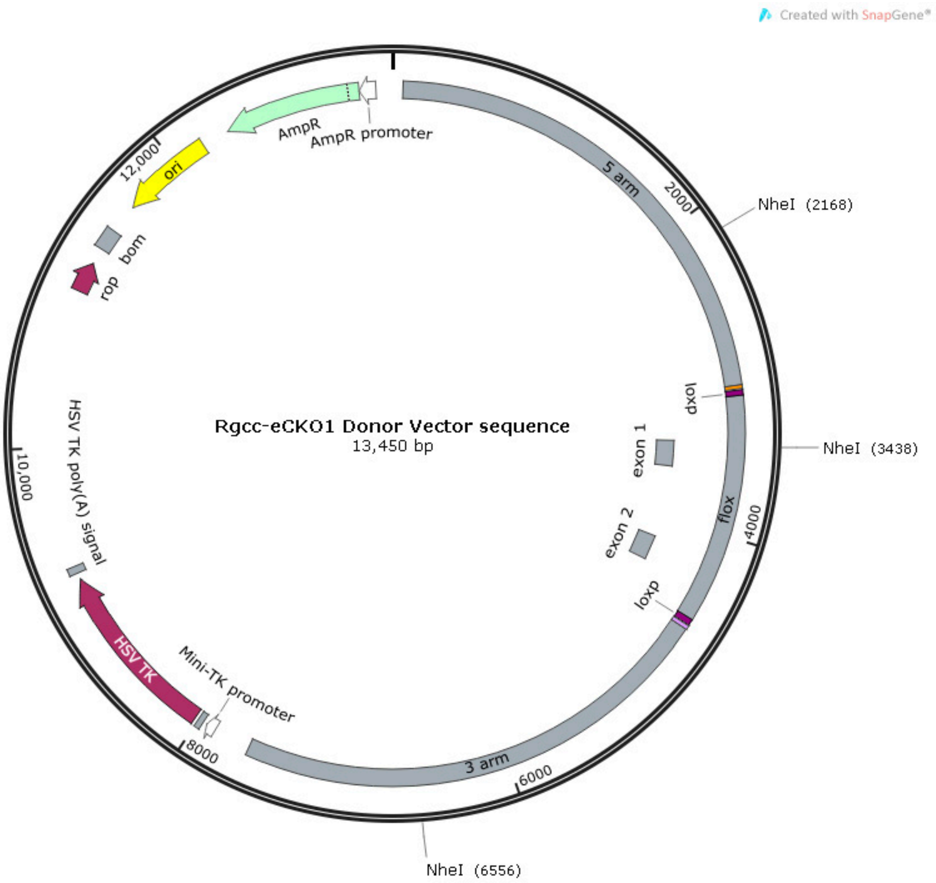

Figure S2: Atlas of homologous recombination vector plasmids

S4.2 Restriction enzyme digestion and identification of homologous recombination plasmids

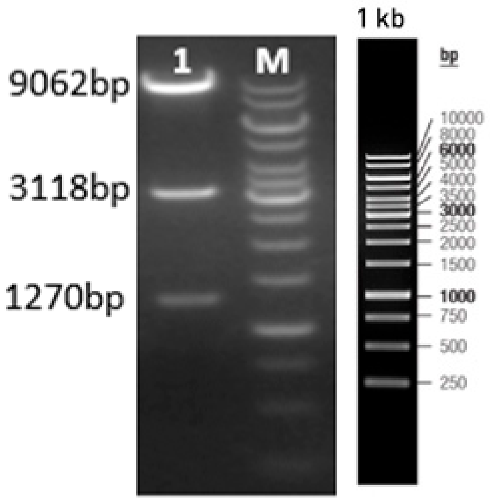

Figure S3: Electrophoretic band pattern of homologous recombination vector after restriction enzyme digestion. 1: Results of NheI digestion; theoretical band sizes are 9062 bp, 3118 bp, and 1270 bp; M: 1 kb DNA ladder.

S4.3 Genotype Identification of F0 Generation Mice

The fertilized embryos after injection were transferred into pseudopregnant female mice, and the mice born approximately 20 days later constituted the F0 generation. Their genotypes were identified through PCR amplification and sequencing. Due to the rapid early cleavage rate of fertilized eggs, the obtained F0 mice are chimeric and may not possess stable genetic traits; thus, further passages are required to obtain F1 mice with stable genetics.

S4.3.1 Identification Strategy for F0 Generation Mice

The identification strategy for F0-generation mice is illustrated in the figure below:

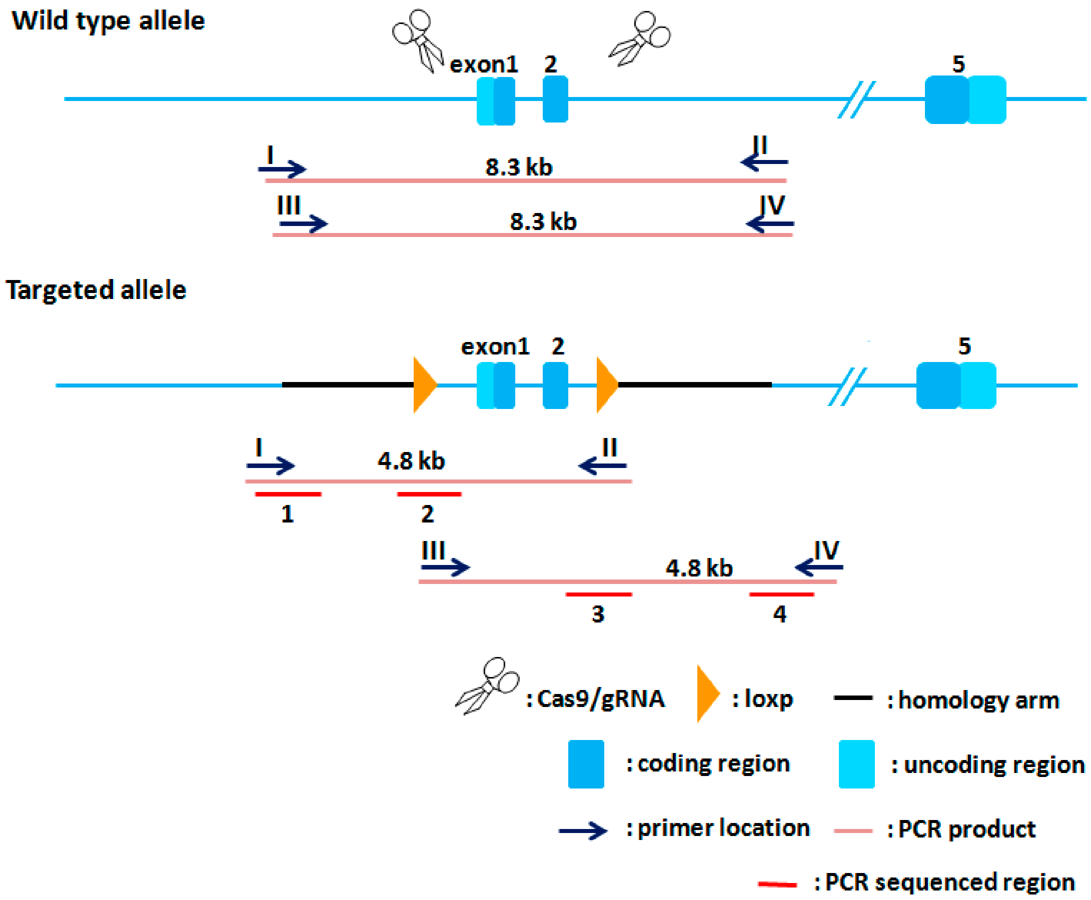

Figure S4: Schematic diagram of identification strategy for F0-generation mice

PCR identification protocol for homologous recombination-positive mice:

For genomes positive for 5′ arm homologous recombination, a 4.8 kb fragment should be amplified; for negative genomes, an 8.3 kb fragment should be amplified. Similarly, for genomes positive for 3′ arm homologous recombination, a 4.8 kb fragment should be amplified; for negative genomes, an 8.3 kb fragment should be amplified.

S4.3.2 PCR identification results of homologous recombination-positive F0 generation mice

The F0 generation mice with positive homologous recombination in both arms were identified as lines 12 and 49; the electrophoretic results of long-fragment PCR identification are shown in Figure S5.

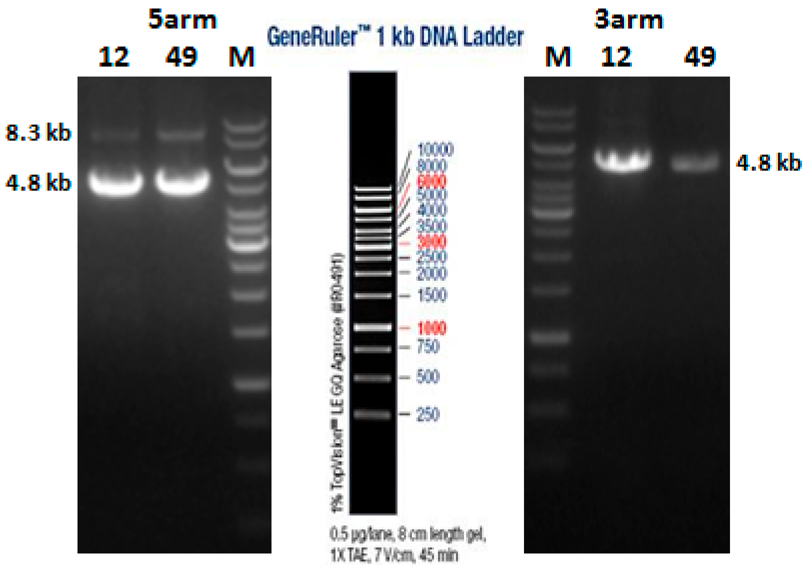

Figure S5: PCR identification electrophoretic pattern of homologous recombination-positive F0-generation mice. Numbers: F0-generation mouse numbers; WT: wild-type control; M: 1 kb DNA marker.

S4.3.3 PCR Identification Method for F0 Generation Mice with Positive 5' Homologous Arm Recombination

Primer Information:

Primer	Sequence 5' --> 3'	Primer Type
I	CAGGCCCTGACGGCTTATT	Forward
II	ACGGACACATGCTACCCTGA	Reverse

reaction system ：

Reaction Component	Volume (μl)
ddH2O	13.2
GXL PCR Buffer	2
2.5 mM dNTP	2
Primer I (10pmol/μl)	0.5
Primer II (10pmol/μl)	0.5
GXL DNA Polymerase*	0.8
genomic DNA	1
Total	20

* PrimeStar GXL （TaKaRa，Code No：R050A）

reaction condition ：

Step #	Temp (℃)	Time	Note
1	94	3 min	-
2	98	15 sec	-
3	61	15 sec	-
4	68	4 min	repeat steps 2-4 for 35cycles
5	68	5 min	-
6	12	-	hold

S4.3.4 PCR Identification Method for F0 Generation Mice with Positive 3′ Homologous Arm Recombination

Primer Information:

Primer	Sequence 5' --> 3'	Primer Type
III	GCAGCTCAGCTATGGACAGAT	Forward
IV	GGTTTCAACTCCCGACTCCT	Reverse

reaction system ：

Reaction Component	Volume (μl)
ddH2O	13.2
GXL PCR Buffer	2
2.5 mM dNTP	2
Primer III (10pmol/μl)	0.5
Primer IV (10pmol/μl)	0.5
GXL DNA Polymerase*	0.8
genomic DNA	1
Total	20

* PrimeStar GXL （TaKaRa，Code No：R050A）

reaction condition ：

Step #	Temp (℃)	Time	Note
1	94	3 min	-
2	98	15 sec	-
3	61	15 sec	-
4	68	4 min	repeat steps 2-4 for 35cycles
5	68	5 min	-
6	12	-	hold

S4.4 Acquisition and Genotypic Identification of F1 Generation Mice

F0-generation positive mice were crossed with wild-type C57BL/6J mice to obtain F1-generation mice, whose genotypes were identified using PCR and sequencing methods.

S4.4.1 PCR identification of the 5 'and 3' homologous arms in F1-generation mice

The PCR identification strategies and methods are identical to those described in Section 4.3 for F0-generation mice. The electrophoretic results of PCR analysis on the 5′ and 3′ homologous arms of F1-generation mice are shown in Figure S6. The mice

identified as positive by PCR were numbered 1, 2, 3, 5, 7, and 9; all were confirmed positive upon sequencing.

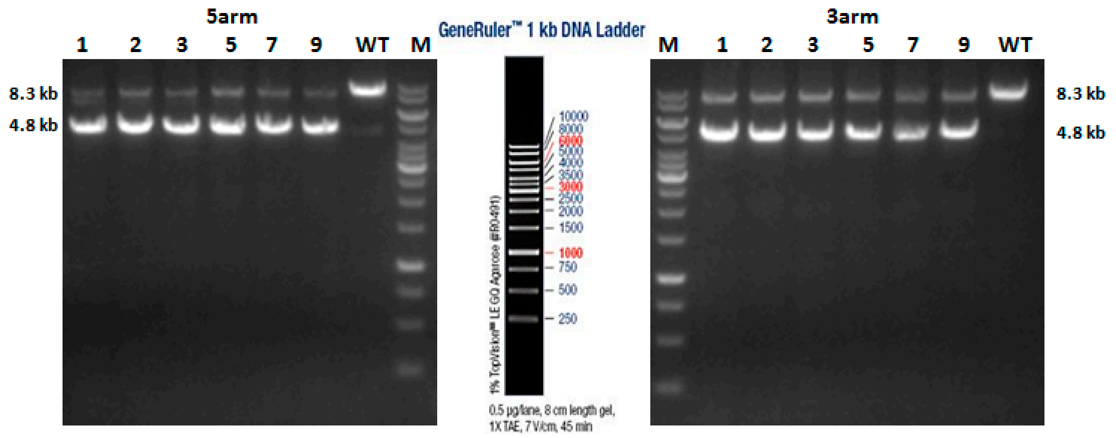

Figure S6: Electrophoretic band pattern of PCR identification of the 5′ homologous arm and 3′ homologous arm in F1-generation mice.

(Numbers: F1 generation mouse identification numbers; WT: wild-type control; M: 1 kb DNA ladder)

S4.4.2 PCR-based identification results and sequence alignment data for F1-generation mice

The PCR identification products from F1-generation positive mice were subjected to sequencing, with a total of 4 sequencing reactions performed. The regions corresponding to these sequencing reactions are shown in Figure S7. For the 5′ homologous arm identification, two sequencing reactions were conducted on the PCR products, labeled as: 1 and 2; for the 3′ homologous arm identification, two sequencing reactions were also conducted on the PCR products, labeled as: 3 and 4.

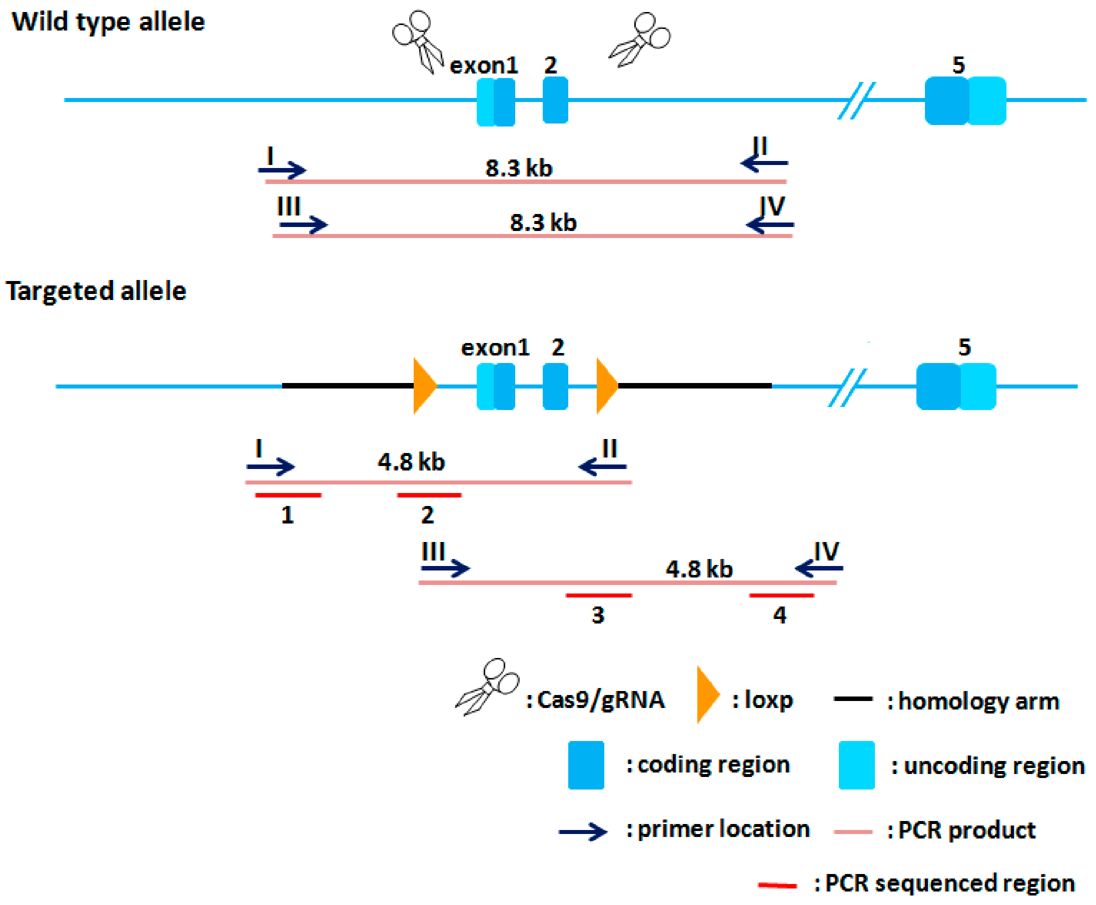

Figure S7: Schematic diagram of PCR product sequencing validation for F1-generation mice.

For the alignment results between the sequencing data and the target sequence, please refer to Sections 5.1.1 and 5.1.2.

S4.4.3 Identification and basic information of F1 generation positive mice

The numbering and basic information of F1-generation positive mice are presented in Table S2.

Table S2: Basic information of F1 generation positive mice

Mouse Number	date of birth	algebra	sex	type	genotype	parental generation	
Mice ID	DOB	Gener-ations	Sex	Type	Genotype	Father	Mother
1	2020/10/1	F1	♂	CKO	He	WT	12#49#
2	2020/10/1	F1	♂	CKO	He	WT	12#49#
3	2020/10/1	F1	♂	CKO	He	WT	12#49#

5	2020/10/1	F1	♀	CKO	He	WT	12#49#
7	2020/10/1	F1	♀	CKO	He	WT	12#49#
9	2020/10/1	F1	♀	CKO	He	WT	12#49#

S5. Sequencing Result Analysis

S5.1 Sequencing results of PCR-amplified products from F1-generation mice and alignment results with the target sequence

The sequencing alignment results are illustrated using mouse strain 1 as a positive control example.

S5.1.1 PCR identification and sequencing alignment results for the 5′ homologous arm of F1-generation mice

1# Sequencing reaction alignment results:

```
Query  3979  TCAGGCCCTGACGGCTTATTTTATTTTTCCCCTGTAGATGTCAGTTACCGATAGGGTTGG  4038
             ||||||||||||||||||||||||||||||||||||||||||||||||||||||||||||
Sbjct  396   TCAGGCCCTGACGGCTTATTTTATTTTTCCCCTGTAGATGTCAGTTACCGATAGGGTTGG  337

Query  4039  TTAACATAGCTGCTTCATGACATCAGAGACCCAAGACACTTCTATGTTTCTGCTGCCATT  4098
             ||||||||||||||||||||||||||||||||||||||||||||||||||||||||||||
Sbjct  336   TTAACATAGCTGCTTCATGACATCAGAGACCCAAGACACTTCTATGTTTCTGCTGCCATT  277

Query  4099  CTAGGAAAGTGATCTCTACCTCATGGCTAAAATCCAGCTTCAAGGCTGCCTGTCATCCTG  4158
             ||||||||||||||||||||||||||||||||||||||||||||||||||||||||||||
Sbjct  276   CTAGGAAAGTGATCTCTACCTCATGGCTAAAATCCAGCTTCAAGGCTGCCTGTCATCCTG  217

Query  4159  TAGGAGAAAGCCAAGTGAGGGAGACTTACTCAGGAAGCCACATAGGAGAGCTCTGTCTGC  4218
             ||||||||||||||||||||||||||||||||||||||||||||||||||||||||||||
Sbjct  216   TAGGAGAAAGCCAAGTGAGGGAGACTTACTCAGGAAGCCACATAGGAGAGCTCTGTCTGC  157

Query  4219  TGCCCAAAATTTAGTCATATCACCAATTTTAACTCAAGATAGACTGGAAATATACTGTTT  4278
             ||||||||||||||||||||||||||||||||||||||||||||||||||||||||||||
Sbjct  156   TGCCCAAAATTTAGTCATATCACCAATTTTAACTCAAGATAGACTGGAAATATACTGTTT  97

Query  4279  CGGGAATGCATGAGTCTACTTGTTTGTTTACAGATTCAACTAGAAGCATCTTCCATAGAG  4338
             ||||||||||||||||||||||||||||||||||||||||||||||||||||||||||||
Sbjct  96    CGGGAATGCATGAGTCTACTTGTTTGTTTACAGATTCAACTAGAAGCATCTTCCATAGAG  37
```

The "Query" field specifies the target sequence (RGC-32-eCKO1 recombinated genomic DNA sequence), the "Subject" field specifies the sequencing results, and the red underline indicates the 5′ arm homologous arm sequence.

2# Sequencing reaction alignment results:

```
Query  7057  ACGCACAACCTTATAAATTCCTTGAGAAAAGTCAGATGGAGGACTGGTTCTTTGCAGAGG  7116
             ||||||||||||||||||||||||||||||||||||||||||||||||||||||||||||
Sbjct  67    ACGCACAACCTTATAAATTCCTTGAGAAAAGTCAGATGGAGGACTGGTTCTTTGCAGAGG  126

Query  7117  CTACTGAGGCCCTTGGCTCCTTTATTTTTGCAGCAAACAAGGAGGCTTTCTGCATGTGCA  7176
             ||||||||||||||||||||||||||||||||||||||||||||||||||||||||||||
Sbjct  127   CTACTGAGGCCCTTGGCTCCTTTATTTTTGCAGCAAACAAGGAGGCTTTCTGCATGTGCA  186

Query  7177  GTTCCATTTCATAGATGAGGCTCAAGGAGGCTCAGCCAGGCAGCTCAGCTATGGACAGAT  7236
             ||||||||||||||||||||||||||||||||||||||||||||||||||||||||||||
Sbjct  187   GTTCCATTTCATAGATGAGGCTCAAGGAGGCTCAGCCAGGCAGCTCAGCTATGGACAGAT  246

Query  7237  GGATATCCATAACTTCGTATAATGTATGCTATACGAAGTTATAAGGCGGGAAACAGGGTA  7296
             ||||||||||||||||||||||||||||||||||||||||||||||||||||||||||||
Sbjct  247   GGATATCCATAACTTCGTATAATGTATGCTATACGAAGTTATAAGGCGGGAAACAGGGTA  306
```

The "Query" field specifies the target sequence (RGC-32-eCKO1 recombinated genomic DNA sequence), while the "Subject" field contains the sequencing results; the red boxed bases represent the LoxP sequence, and the red underline denotes the 5′ arm homologous arm sequence.

S5.1.2 PCR-based identification and sequencing alignment results for the 3′ homologous arms of F1-generation mice

3# Sequencing reaction alignment results:

```
Query  8701  GGTAATAACTTCGTATAATGTATGCTATACGAAGTTATGGGGTACCCCTCAGGGTAGCAT  8760
             ||||||||||||||||||||||||||||||||||||||||||||||||||||||||||||
Sbjct  204   GGTAATAACTTCGTATAATGTATGCTATACGAAGTTATGGGGTACCCCTCAGGGTAGCAT  145

Query  8761  GTGTCCGTCGAGGGTGGGTGGGTGGCTCTTAAAGAGCTG-TGGGTCTACCTGTCACGGCA  8819
             ||||||||||||||||||||||||||||||||||||| | ||||||||||||||||||||
Sbjct  144   GTGTCCGTCGAGGGTGGGTGGGTGGCTCTTAAAGAGCAGGTGGGTCTACCTGTCACGGCA  85

Query  8820  GGTGTGTACCATGAGGCCTGAGGGTTTGTGCAGTCAGCCAGAACTCCTGGGAGTCCAGCC  8879
             ||||||||||||||||||||||||||||||||||||||||||||||||||||||||||||
Sbjct  84    GGTGTGTACCATGAGGCCTGAGGGTTTGTGCAGTCAGCCAGAACTCCTGGGAGTCCAGCC  25
```

The "Query" field specifies the target sequence (RGC-32-eCKO1 recombinated genomic DNA sequence), the "Subject" field contains the sequencing results, the red boxed bases represent the LoxP sequence, and the red underline denotes the 3'-arm homologous arm sequence.

4# Sequencing reaction alignment results:

```
Query  11678  TTCTACTGGTTGACTTCGTAAATGGTTTATTATACTGACTTATGCCTTTAAAGATGAGGC  11737
              ||||||||||||||||||||||||||||||||||||||||||||||||||||||||||||
Sbjct  15     TTCTACTGGTTGACTTCGTAAATGGTTTATTATACTGACTTATGCCTTTAAAGATGAGGC  74

Query  11738  TGAGGTGTGATTCTTATATATTAGGGTTTCACATAAGTCATCCTGTGAGAGGACAAGTGT  11797
              ||||||||||||||||||||||||||||||||||||||||||||||||||||||||||||
Sbjct  75     TGAGGTGTGATTCTTATATATTAGGGTTTCACATAAGTCATCCTGTGAGAGGACAAGTGT  134

Query  11798  GCTGCTGTTTAATAGGAAAGGTCTGAGGTCTACTGTCCATGCCGTAGCTATGATAAGATT  11857
              ||||||||||||||||||||||||||||||||||||||||||||||||||||||||||||
Sbjct  135    GCTGCTGTTTAATAGGAAAGGTCTGAGGTCTACTGTCCATGCCGTAGCTATGATAAGATT  194

Query  11858  GTAGATATTTGGGGAGAATAGGGACTGGGTGCTTTAGCATAGCAACACTTAGAACATCTA  11917
              ||||||||||||||||||||||||||||||||||||||||||||||||||||||||||||
Sbjct  195    GTAGATATTTGGGGAGAATAGGGACTGGGTGCTTTAGCATAGCAACACTTAGAACATCTA  254

Query  11918  CTTATGTTTAGAATATGCTGGTCTGGCTCTGAGAGAGGAGACCTTAGGAATCCTAGAAAC  11977
              ||||||||||||||||||||||||||||||||||||||||||||||||||||||||||||
Sbjct  255    CTTATGTTTAGAATATGCTGGTCTGGCTCTGAGAGAGGAGACCTTAGGAATCCTAGAAAC  314

Query  11978  CAGAATGACAGTTACCAAGCCAGTGGCTAATGGAGCTGAGGAGTCGGGAGTTGAAACC  12035
              ||||||||||||||||||||||||||||||||||||||||||||||||||||||||||
Sbjct  315    CAGAATGACAGTTACCAAGCCAGTGGCTAATGGAGCTGAGGAGTCGGGAGTTGAAACC  372
```

The "Query" field specifies the target sequence (RGC-32-eCKO1 recombinated genomic DNA sequence), the "Subject" field specifies the sequencing results, and the red underline indicates the 3-arm homologous arm sequence.
