# Supplementary material for: Tubule-Specific RGC-32 Knockout Exhibits Direct and Progressive Aggravating Activity Against Renal Function in an Ischemia–Reperfusion Mouse Model
Source: Biology (Basel). 2026 Sep 4;15(17):1535. doi: 10.3390/biology15171535 (PMC13565509; doi:10.3390/biology15171535)
Supplement: Supplementary file 1 [file biology-15-01535-s001.zip › Supplementary Material S2-Western blot.pdf]

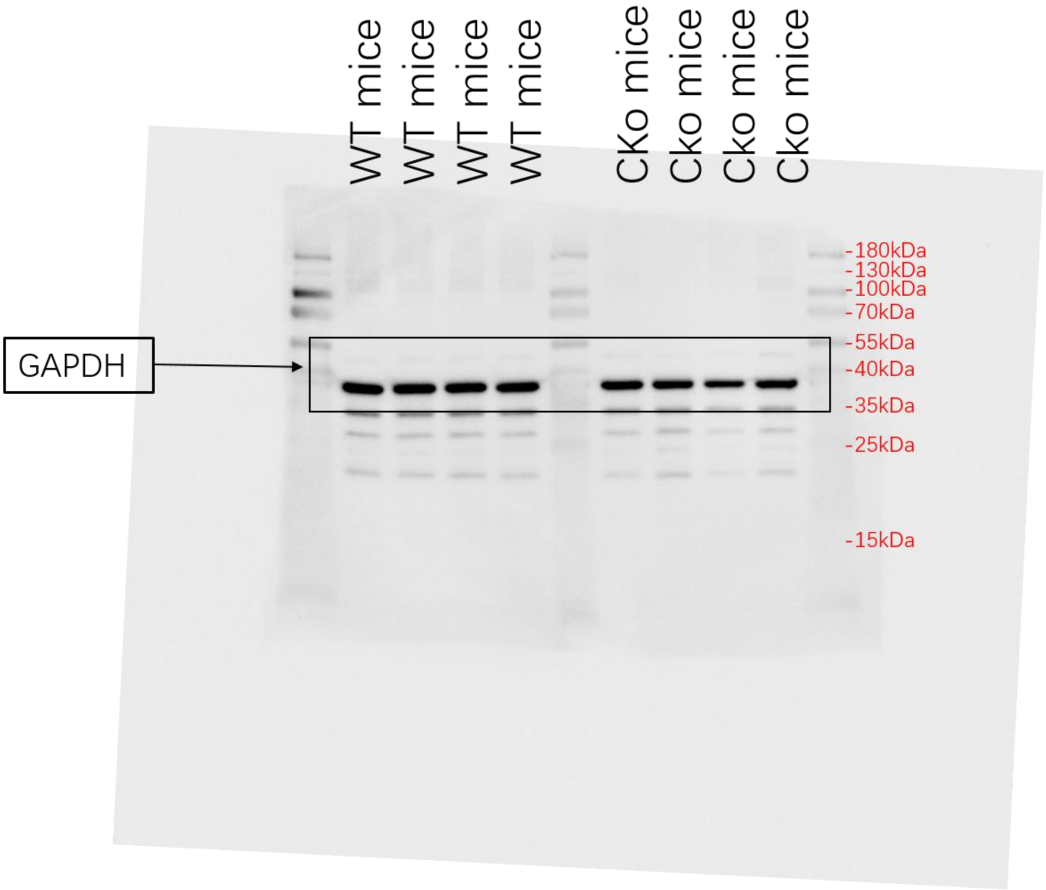

Western blot membrane of GAPDH (~36 kDa) protein detected with anti-GAPDH (ET1601-4; 1: 50000; HUABIO) antibody.

Gel-separated proteins were transferred to PVDF by wet electroblotting (300mA, 30min).

Membranes, incubated with a horseradish peroxidase-conjugated secondary antibody (HA1001;1:50,000~1:100,000; HUABIO), were developed with Pierce™ ECL Western Substrate Kit (Thermo Fisher Scientific).

Weight marker (molecular weight in kDa): Thermo Scientific™/PageRuler™ Prestained Protein Ladder, 10 to 180 kDa; catalogue number: 26616.

The gray value of target protein bands was quantified using ImageJ2 software (ImageJ2 v.2.14.0/1.54f). Briefly, the rectangular selection tool was used to frame each protein band with identical area; the integrated density (IntDen, integrated gray value) of each band was detected. The gray value of target protein was normalized to the corresponding internal reference protein (GAPDH) to obtain the relative protein expression level. Four independent biological replicates were performed for statistical analysis. The gray values are as follows:

1	36411.175
2	37211.418
3	35654.761
4	36455.882
5	32013.004
6	28796.004
7	21770.983
8	28456.296

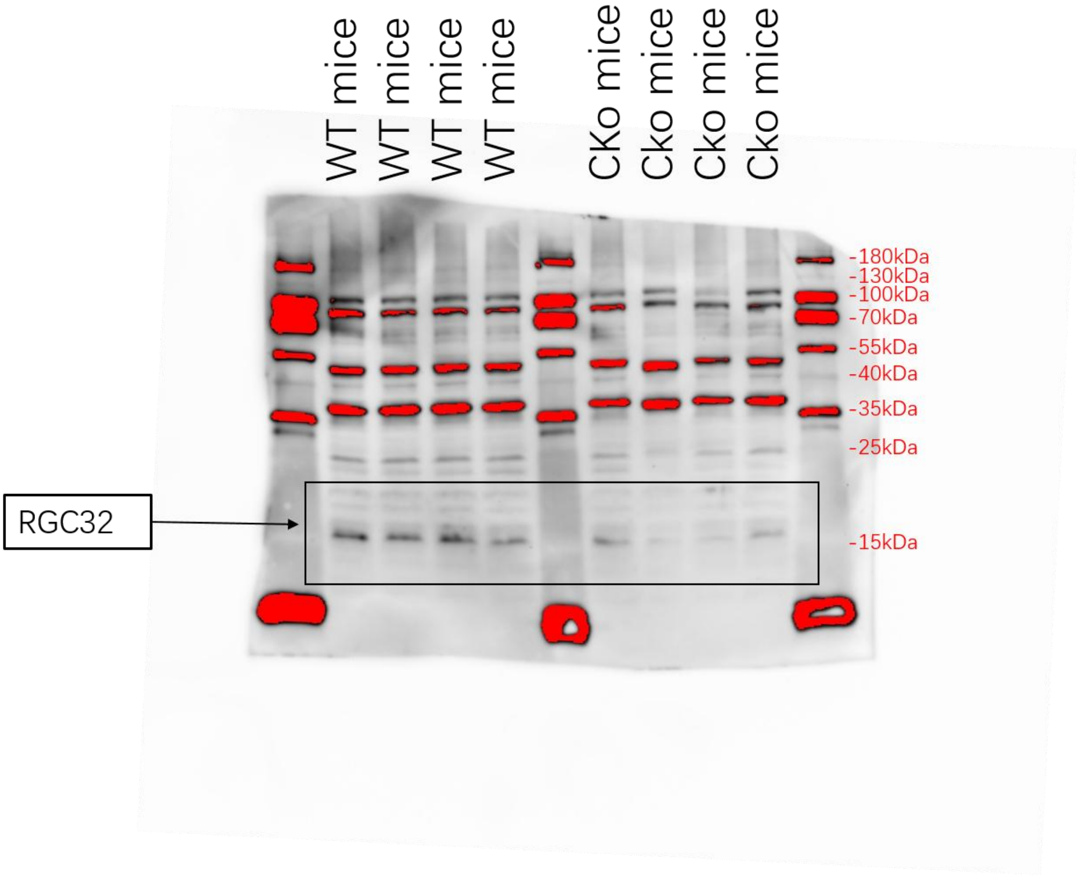

Western blot membrane of RGC32 (~15 kDa) protein detected with anti- RGC32 (sc-84223; 1: 3000; santacruze) antibody.

Gel-separated proteins were transferred to PVDF by wet electroblotting (300mA, 30min).

Membranes, incubated with a horseradish peroxidase-conjugated secondary antibody (HA1001;1:50,000~1:100,000; HUABIO), were developed with Pierce™ ECL Western Substrate Kit (Thermo Fisher Scientific).

Weight marker (molecular weight in kDa): Thermo Scientific™/PageRuler™ Prestained Protein Ladder, 10 to 180 kDa; catalogue number: 26616.

The gray value of target protein bands was quantified using ImageJ2 software (ImageJ2 v.2.14.0/1.54f). Briefly, the rectangular selection tool was used to frame each protein band with identical area; the integrated density (IntDen, integrated gray value) of each band was detected. The gray value of target protein was normalized to the corresponding internal reference protein (GAPDH) to obtain the relative protein expression level. Four independent biological replicates were performed for statistical analysis. The gray values are as follows:

1	27409.200
2	26209.200
3	35898.513
4	23042.765
5	17597.016
6	2834.477
7	3901.841
8	6122.196

	Lane	RGC-32	GAPDH	RGC-32/GAPDH
**WT**	1	27409.200	36411.175	**0.7528**
	2	26209.200	37211.418	**0.7043**
	3	35898.513	35654.761	**1.0068**
	4	23042.765	36455.882	**0.6321**
**CKO**	5	17597.016	32013.004	**0.5497**
	6	2834.477	28796.004	**0.0984**
	7	3901.841	21770.983	**0.1792**
	8	6122.196	28456.296	**0.2151**
